# Satralizumab in the management of aquaporin-4 antibody-positive neuromyelitis optica spectrum disorder during pregnancy: a case report

**DOI:** 10.3389/fimmu.2026.1833243

**Published:** 2026-05-21

**Authors:** Man Ding, Bo Yin, Jiajia Yao, Yin Liu, Hongjuan Dong, Zuneng Lu

**Affiliations:** Department of Neurology, Renmin Hospital of Wuhan University, Wuhan, China

**Keywords:** aquaporin-4, case report, neuromyelitis optica spectrum disorder, pregnancy, satralizumab

## Abstract

We report the case of a 25-year-old pregnant woman who was diagnosed with aquaporin-4 antibody-positive (AQP4+) neuromyelitis optica spectrum disorder (NMOSD) at 19 weeks of gestation. During the acute stage of the disease, she was treated with high-dose prednisone pulse therapy. Beginning in the fourth week after disease onset, satralizumab was introduced as an adjunctive therapy, which enabled the progressive reduction and eventual discontinuation of corticosteroids. Subsequent follow-up showed marked improvement in visual field defect, successful spontaneous delivery, stable maternal condition, and transient early neonatal complications with favorable short-term recovery. This represents the first reported case in China of a patient diagnosed with AQP4 + NMOSD during pregnancy who was treated with satralizumab. This case provides additional clinical evidence to inform therapeutic decision-making for NMOSD occurring in pregnancy.

## Background

Neuromyelitis optica spectrum disorder (NMOSD) is an autoimmune inflammatory demyelinating disease of the central nervous system that primarily affects the optic nerves, spinal cord, and brain. Aquaporin-4 immunoglobulin G (AQP4-IgG) serves as the key disease-specific biomarker. Immunological adaptations during pregnancy may increase the risk of NMOSD relapse, whereas conventional immunosuppressive agents, including azathioprine and mycophenolate mofetil, are associated with definite teratogenic potential. Satralizumab is a humanized IgG1 monoclonal antibody targeting the interleukin-6 (IL-6) receptor with relatively low placental transfer and is therefore theoretically safer for use during pregnancy; however, real-world evidence in pregnant patients with NMOSD remains limited. This case report describes the complete clinical course and maternal-neonatal outcomes of satralizumab therapy in a pregnant woman with AQP4 antibody-positive NMOSD. By reviewing the disease progression and treatment strategy, this report aims to provide clinical reference for the management of such rare cases.

## Case presentation

A 25-year-old woman at 19 + 5 weeks of gestation was admitted on September 13, 2024, with a 11-day history of acute visual decline in the right eye. On September 2, 2024, she experienced blurred vision in the right eye of unknown origin, which progressively deteriorated to marked visual impairment, without accompanying neurological manifestations. She first sought care at a local ophthalmology department, where examination identified a superior nasal visual field defect in the right eye and a positive serum AQP4 antibody result. She was subsequently referred to our neurology department for further assessment.

Her medical history was notable for an ongoing 19-week intrauterine pregnancy. In 2019, she was reportedly hospitalized at another hospital for right-sided retrobulbar optic neuritis and received approximately two weeks of treatment, with complete visual recovery thereafter. However, the original medical records were unavailable, and the specific treatment regimen could not be verified. No other significant medical history was reported. On admission, she was alert and oriented, with normal mental status and fluent speech. Visual acuity in the right eye was limited to light perception. Pupils were equal and round, approximately 3 mm in diameter. A relative afferent pupillary defect was noted in the right eye. There was no evidence of nystagmus or diplopia. The remainder of the neurological and systemic examinations was unremarkable.

**Figure 1:**
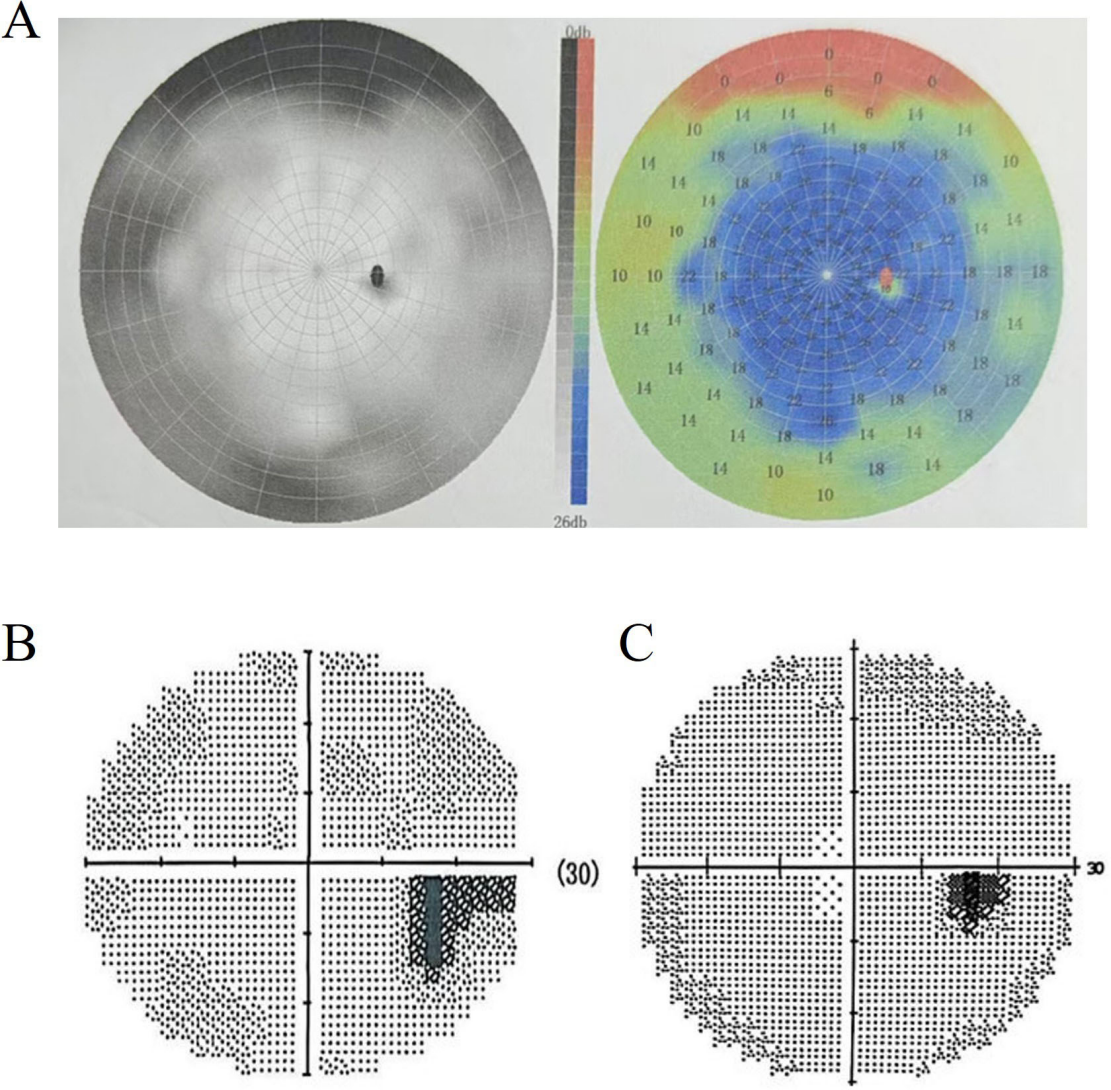
Visual field defect in the right eye (RE) and improvement after treatment. **(A)** Visual field examination demonstrated markedly reduced retinal sensitivity across all four quadrants. Mean sensitivities were 4.61 dB (quadrant I), 4.33 dB (quadrant II), 3.67 dB (quadrant III), and 3.33 dB (quadrant IV). **(B)** Dec 19, 2024: Visual field test shows a superior nasal quadrant defect in the RE (represented by the black shaded area). **(C)** May 31, 2025: The extent of the visual field defect (black shaded area) in the RE has diminished.

Additional investigations showed a right superior nasal quadrant visual field defect with reduced visual acuity on ophthalmologic evaluation. The retinal sensitivity was severely reduced in all quadrants (3.33–4.61 dB), consistent with diffuse visual field loss (**Figure 1A**), based on automated perimetry performed on September 6, 2024. Visual evoked potentials, performed on September 7, 2024, were within normal limits, whereas the N75-P100 amplitude was relatively reduced in the right eye compared with the contralateral eye. The Expanded Disability Status Scale (EDSS) score at presentation was 3 with visual functional system score of 4-5. Serum AQP4 antibody titer was 1:3200 (test method: Cell-Based Assay). Brain magnetic resonance imaging (MRI) on admission demonstrated no abnormal signal changes. Taken together, the clinical history, clinical manifestations, visual field loss, and positive serum AQP4 antibodies supported a diagnosis of NMOSD. Obstetric ultrasonography confirmed a 19+ week intrauterine pregnancy, fetal tachycardia, and normal cervical length. Immunological testing revealed IgG 8.42 g/L (reference 8.6-17.4 g/L); CD4+ T cells 30.73% (reference 33-58%); B cells 30.21% (reference 5-22%); absolute B-cell count 832/μL (reference 80-616/μL); and NK cells 4.67% (reference 5-26%).

**Figure 2:**
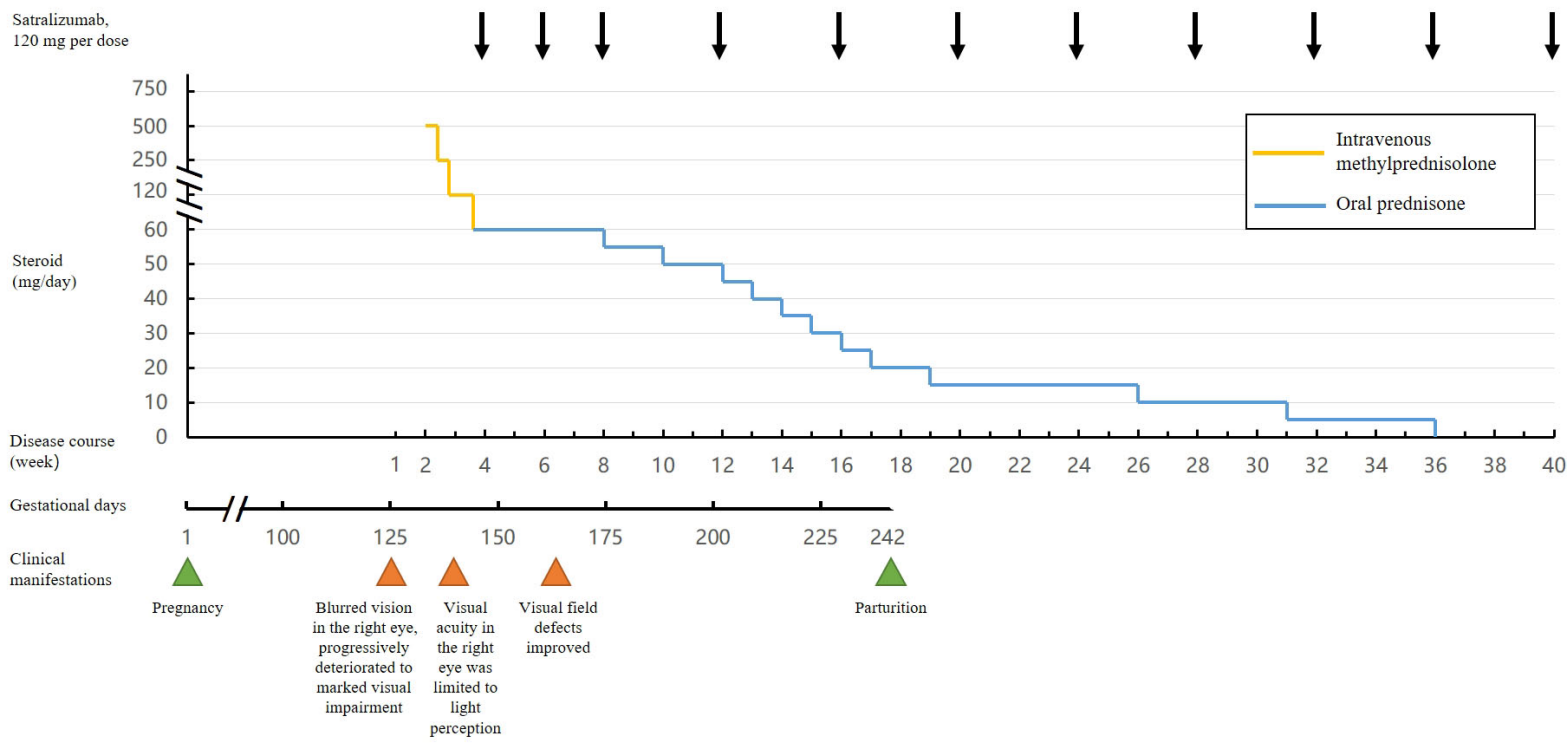
Timeline of the patient’s treatment course and medication administration.

Beginning on September 13, 2024, the patient underwent intravenous methylprednisolone pulse therapy for the acute attack, administered at 500 mg/day for 3 days, followed by 250 mg/day for 3 days and 120 mg/day for 5 days. On September 24, 2024, the patient was transitioned to oral prednisone at 60 mg/day, which was maintained for one month (**Figure 2**). On September 26, 2024, at 21 + 4 weeks of gestation, after comprehensive counseling regarding the potential risks of satralizumab, including infection-related maternal and fetal mortality, and after obtaining written informed consent, subcutaneous satralizumab 120 mg was administered as the initial dose. The second and third 120 mg injections were given at 2 and 4 weeks following the first administration. By November 13, 2024, the serum AQP4 antibody titer had declined to 1:320 (test method: Cell-Based Assay). The corticosteroid dose was subsequently reduced in a stepwise manner and completely discontinued on May 8, 2025. The patient has continued to receive maintenance therapy with satralizumab at a dose of 120 mg every 4 weeks, and is still undergoing treatment. The last follow-up date in this report was January 2026. Visual field defects improved following treatment (**Figures 1B, C**). A follow-up MRI performed on June 2, 2025, revealed no abnormal findings. Clinically, the patient remained stable with an EDSS score of 1, visual functional system score of 1, and resumed normal occupational and daily activities.

**Figure 3:**
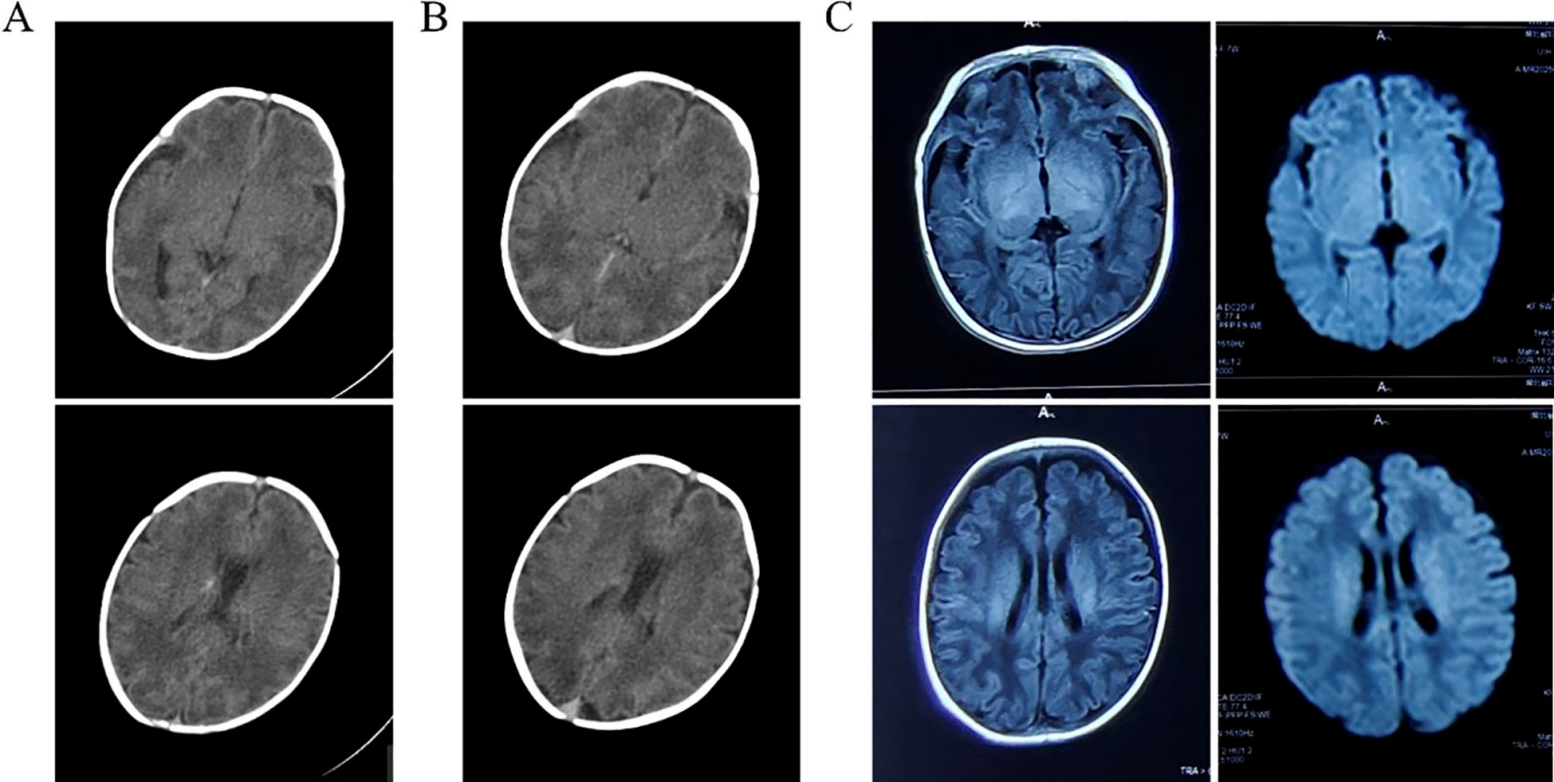
Serial neuroimaging findings in the neonate. **(A)** Cranial CT on postnatal day 4 showed extensive symmetric patchy hypodensities in the bilateral frontotemporal, parietal, and occipital white matter, consistent with diffuse cerebral edema, with a focal hyperdensity in the right caudate nucleus region suggestive of associated germinal matrix and/or low-grade intraventricular hemorrhage. **(B)** Repeat cranial CT on postnatal day 9 at the same axial level showed partial resolution of the white matter hypodensities and reduction of the right caudate region hyperdensity, indicating improvement of cerebral edema and hemorrhage absorption. **(C)** Brain MRI at approximately 2 months of age showed no diffusion restriction or definite chronic ischemic lesions, with only minimal residual hemorrhagic change adjacent to the right lateral ventricle.

She delivered spontaneously at a late preterm gestational age (34 + 4 weeks) on December 24, 2024. This preterm female infant was admitted to the neonatal ward at 24 minutes of life because of cyanosis, respiratory distress, lethargy, hypotonia, and diminished primitive reflexes. On admission, the neonate’s temperature was 35.0 °C, respiratory rate 48 breaths/min, heart rate 136 beats/min, and capillary blood glucose 1.3 mmol/L. The original delivery records did not provide formal 1- and 5-minute Apgar scores; based on the neonatal condition documented at 24 minutes after birth, the infant appeared to have experienced impaired early postnatal adaptation, with retrospectively estimated Apgar scores of approximately 4–5 at 1 minute and 5–6 at 5 minutes. Cranial CT performed on postnatal day 4 revealed diffuse symmetric white matter hypodensity involving the bilateral frontal, temporal, parietal, and occipital regions (approximately 17 HU), consistent with transient cerebral white matter edema/injury, together with a small focal hyperdense lesion in the right caudate region suggestive of a low-grade germinal matrix/subependymal hemorrhage (**Figure 3A**). No midline shift or ventricular enlargement was present. Considering both the imaging findings and clinical presentation, the neonate was diagnosed with hypoxic-ischemic encephalopathy. Follow-up CT on postnatal day 8 demonstrated partial resolution of the white matter hypodensity (17–21 HU) and gradual resorption of the hemorrhagic focus, with preserved ventricular configuration and normal midline alignment (**Figure 3B**). The infant was discharged in stable condition on the same day. At approximately 2 months of age, follow-up brain MRI showed no acute or chronic ischemic lesions, no diffusion restriction, and no major residual parenchymal injury; only minimal residual right subependymal hemorrhagic change was suspected. Myelination was appropriate for corrected gestational age, and ventricular size, cortical sulcation/gyration, and midline structures were normal (**Figure 3C**). These serial imaging findings suggest a favorable short-term neuroimaging outcome, with largely reversible early abnormalities and no evidence of major persistent structural brain injury. Although breastfeeding was discussed, the patient elected to use formula feeding. At the most recent follow-up in January 2026 (approximately 13 months after birth), the parents reported that the child had achieved age-appropriate developmental milestones, with no apparent delay in motor or language development, and functional abilities generally comparable to those of peers of the same age. Formal standardized neurodevelopmental assessment was not available.

## Discussion

NMOSD is an immune-mediated inflammatory disease of the central nervous system characterized by frequent relapses and substantial risk of disability. NMOSD occurring during pregnancy is uncommon and is generally regarded as a high-risk condition. Physiological hormonal and immunological alterations in pregnancy may promote a shift from Th1-dominant to Th2-dominant immune responses. Concurrent activation of the interleukin-6 (IL-6) pathway and disruption of the Treg/Th17 balance may further aggravate disease activity in NMOSD (1, 2).

Currently, there are no standardized guidelines for the management of NMOSD during pregnancy. In clinical practice, glucocorticoids are typically administered for acute exacerbations, whereas azathioprine, mycophenolate mofetil, or rituximab are used for maintenance therapy. However, robust large-scale data regarding their safety and effectiveness in pregnant patients with NMOSD remain lacking, and some conventional immunosuppressive therapies may be limited by potential fetal risks or the need for treatment interruption during pregnancy. Therefore, effective and relatively safe targeted therapies are needed in this clinical setting. The 2023 international Delphi consensus on the management of AQP4-IgG-positive NMOSD (3) indicated that although evidence for biologic agents in pregnant NMOSD patients is limited, studies in pregnant women with rheumatoid arthritis suggest that tocilizumab may be relatively safe. In addition, a recent case report of a young woman with AQP4-IgG-seropositive NMOSD was treated with eculizumab throughout pregnancy with no adverse events and gave birth to a healthy child (4). Patients receiving complement component 5 inhibitor therapies are at risk of meningococcal infection and should receive meningococcal vaccination. Although emerging evidence suggests that certain biologic therapies may be feasible during pregnancy, data on their safety and effectiveness in pregnant patients with NMOSD remain limited.

Satralizumab is a humanized monoclonal antibody directed against the IL-6 receptor and is approved for AQP4-positive NMOSD. By inhibiting IL-6 signaling, it modulates immune responses and helps restore the Th17/Treg equilibrium, providing a theoretical advantage in the peripregnancy setting. Several reports have described successful use of satralizumab in pregnancy-associated NMOSD. Yoshida et al. (5) reported a patient desiring pregnancy who, after repeated treatment with methylprednisolone, intravenous immunoglobulin, and oral prednisolone combined with azathioprine, remained relapse-free but developed moon face and weight gain, necessitating steroid reduction. Considering her reproductive plans and stable disease status, satralizumab was initiated on day 110 after onset. She subsequently delivered successfully, received 34 injections over 2.5 years during pregnancy and lactation, experienced no relapses, reduced prednisolone to 8 mg/day, and the infant showed normal development. Nakashima et al. (6) described a 35-year-old patient with recurrent relapses despite prednisolone and azathioprine; after introduction of satralizumab, the disease stabilized, and she delivered without relapse, with normal fetal growth and no breastfeeding. Yaguchi et al. (7) reported a 30-year-old patient who relapsed around her first delivery despite maintenance prednisolone 20 mg; after addition of satralizumab, further relapses were prevented. At 41 years of age, she conceived again and achieved successful delivery while receiving prednisolone 10 mg and satralizumab throughout pregnancy without relapse. Cord blood satralizumab concentration was approximately half that of maternal serum, and the infant developed normally.

In China, satralizumab has been reported in a patient in late pregnancy with demyelinating optic neuritis who was negative for both AQP4 and MOG antibodies (8). Although that patient did not meet diagnostic criteria for NMOSD, satralizumab was selected because of its comparatively favorable pregnancy safety profile among available biologic therapies. In the present case, the patient was pregnant at onset, presented with visual field defects and visual impairment, and tested positive for AQP4 antibodies, thereby confirming the diagnosis of NMOSD. To our knowledge, this represents the first reported case in China of pregnancy-associated AQP4-positive NMOSD treated with satralizumab, with long-term maternal and neonatal follow-up. Unlike previous planned pregnancies, our patient had unplanned pregnancy and onset during gestation. The extended duration of therapy and follow-up offers meaningful safety observations. Considering the elevated risk of relapse during the peripartum period and the complexity of clinical management, this case provides real-world clinical evidence and underscores the importance of individualized therapeutic strategies.

With respect to neonatal outcomes, this pregnancy was complicated by preterm delivery, low birth weight, and neonatal hypoxic-ischemic encephalopathy. Previous studies have shown that NMOSD during pregnancy is associated with increased risks of adverse obstetric and neonatal outcomes, including miscarriage, prematurity, and low birth weight (9). In addition, prematurity itself is a well-recognized risk factor for neonatal hypoxic-ischemic and hemorrhagic complications. Therefore, although a causal relationship cannot be established in a single case, these neonatal complications may be more plausibly related to the underlying maternal disease and prematurity rather than directly attributable to satralizumab exposure. After supportive neonatal management, the infant’s condition stabilized. At 13 months of follow-up, according to parental report, the child demonstrated generally reassuring developmental progress, with motor and language function considered comparable to those of age-matched peers.

Previously reported Japanese cases involved planned pregnancies in patients with established NMOSD diagnoses prior to initiation of satralizumab therapy. In contrast, our patient experienced an unexpected onset of vision loss during pregnancy and was diagnosed with AQP4-positive NMOSD. Furthermore, we speculate that the patient’s retrobulbar optic neuritis in 2019 may have been a single episode. Pregnancy is a high-risk period for NMOSD. Importantly, not all women are aware of their NMOSD status before conception; Wang et al. (10) reported that nearly one-third of NMOSD pregnancies in China were unplanned. In this case, the introduction of satralizumab enabled discontinuation of corticosteroids, thereby reducing drug side effects and the risks associated with polypharmacy. These findings provide additional clinical evidence supporting the use of satralizumab in pregnant patients with NMOSD and suggest that it may represent a relatively safe therapeutic option for this special population.

We acknowledge certain limitations in this report. Due to the retrospective nature of this case analysis and the unavailability of accessible medical records from the previous treating hospital, some early clinical details, such as the specific treatment regimen during the patient’s first episode in 2019, could not be retrieved. Nevertheless, the clinical course during her most recent pregnancy provides valuable real-world evidence for the use of satralizumab in managing NMOSD in pregnant patients.

## Conclusion

This case describes the use of satralizumab during an unplanned pregnancy in a patient with NMOSD, in whom maternal disease remained clinically stable and concomitant medication use was reduced. Although the neonate developed preterm birth and hypoxic-ischemic encephalopathy, short-term clinical improvement was observed during follow-up. In the context of a complex perinatal course, no clear evidence from this single case supports a direct causal association between satralizumab exposure and the neonatal complications, although such a contribution cannot be definitively excluded. Overall, this report adds limited real-world clinical experience regarding satralizumab exposure during pregnancy, and its findings should be interpreted cautiously. Larger cohort studies and long-term pediatric developmental follow-up are needed to better clarify maternal and neonatal outcomes and the potential clinical role and the safety of satralizumab in this setting.

## Data Availability

The original contributions presented in the study are included in the article/supplementary material. Further inquiries can be directed to the corresponding authors.
